# Assessment of the Prevalence of *Angiostrongylus cantonensis* Infection in Gastropods of Puerto Rico

**DOI:** 10.4269/ajtmh.25-0683

**Published:** 2026-05-28

**Authors:** Julia Marie Stewart, David G. Robinson, Kathleen M. Howe, Argon Steel, Leyinska Wiscovitch, Mildred Sosa, Shirley Cruz, Cosme Guzman, Susan I. Jarvi

**Affiliations:** ^1^ Daniel K. Inouye College of Pharmacy, University of Hawai’i at Hilo, Hilo, Hawaii, USA;; ^2^ Mollusca Global Services, Philadelphia, Pennsylvania, USA;; ^3^ US Department of Agriculture Animal and Plant Health Inspection Service Plant Protection and Quarantine Program, San Juan, Puerto Rico and St Croix, US Virgin Islands

## Abstract

Biological infections of the zoonotic nematode *Angiostrongylus cantonensis* have caused neuroangiostrongyliasis (NAS) in many people around the world and have impacted the neurological health of various wild and domestic animals. Several Caribbean countries have reported cases of NAS caused by the rat lungworm; however, the most recent study in Puerto Rico was in 1984. Here, we conducted quantitative polymerase chain reaction (PCR) to evaluate levels of *A. cantonensis* DNA in 205 gastropod samples from 20 different species across eight sites in Puerto Rico. An overall estimate of 35.6% (*n* = 73/205) was determined to be positive. We found that approximately 67.2% (*n* = 45/67) of *Parmarion intermedius* and* Parmarion martensi* were PCR positive for *A. cantonensis* DNA. *Parmarion* spp. and *Caracolus* spp. samples contained significantly greater concentrations of *A. cantonensis* DNA by several orders of magnitude than all of the other species tested, suggesting higher infection levels. Gastropods collected from three sites on the northwestern side of the island yielded significantly less *A. cantonensis* DNA than midregion sites, suggesting lower infection levels. *Angiostrongylus cantonensis* was found in several areas across Puerto Rico, with the highest nematode prevalence found at somewhat higher elevations (471–532 meters). An extensive temporal study of *A. cantonensis* in gastropods as well as in the definitive rat host would provide a better understanding of the widespread prevalence and biogeography of this parasite in Puerto Rico, and thus, it would provide a better estimate of infection risks to humans and other animals.

## INTRODUCTION

Rat lungworm disease or neuroangiostrongyliasis (NAS) is a tropical infectious disease caused by the nematode *Angiostrongylus cantonensis* (Nematoda: Metastrongylidae).^1^ In humans, it can cause ocular angiostrongyliasis, debilitating neurological issues, and/or eosinophilic meningitis when the larvae (after having reached the brain) generally die. Neuroangiostrongyliasis was first observed in a human case in 1945 in Taiwan, although *A. cantonensis* was not determined to be the causative agent of NAS until 1959.^2^ The completion of the *A. cantonensis* life cycle requires a definitive rat host and an intermediate gastropod host. *Angiostrongylus cantonensis* adults live and reproduce in the pulmonary artery and lungs of many rat species. The first-stage larvae are excreted in rat feces, ingested by aquatic and terrestrial gastropods, and develop to the infective third-stage larvae (L3). They are then ingested by rats, and the cycle starts again.^3^ Although rats are the definitive hosts for the *A. cantonensis* life cycle, other animals may carry *A. cantonensis* and potentially infect humans.^4^^,^^5^ Animals shown to be accidental or paratenic hosts include dogs,^4^ horses,^6^^,^^7^ nonhuman primates,^8^ coqui frogs, cane toads, centipedes,^9^ lizards, and freshwater shrimp (reviewed by Cross^10^ in 2004).

One of the most common modes of infection is accidental or intentional ingestion of gastropods carrying L3 *A. cantonensis* larvae, which are directly infectious to humans and other animals, in either contaminated food or water.^11^ Symptoms in humans include (but are not limited to) headaches, nausea, neck stiffness, vomiting, pain in arms and shoulders, paresthesia, bowel and bladder dysfunction, and hyporeflexia.^12^^–^^16^

Humans have been affected by NAS on a global scale (reviewed in reference 17). In the last few decades, case numbers have increased in locations such as Thailand, Australia, and Hawai’i as well as on the U.S. continent (reviewed in reference 18). An increase in severe cases was observed at the turn of the millennium in Hawai’i, which coincided with the introduction of *Parmarion martensi* (a semislug) from Southeast Asia to east Hawai’i Island^19^ and east Maui.^20^^,^^21^ Much of the spread of both the definitive and intermediate hosts (rats and gastropods, respectively) is thought to have been caused by increased human activity, especially through the ornamental plant trade, climate change, worldwide shipping, and other means of increasing transmission. Although the first cases of NAS occurred in Asia and then, in the Pacific basin, three autochthonous pediatric cases were reported recently in the United States in Florida in 2021 and 2022.^22^ Neuroangiostrongyliasis has also affected people throughout the Caribbean over the past few decades,^10^ including people in Jamaica,^23^^–^^25^ Martinique,^26^ Guadeloupe,^27^ Cuba,^28^^–^^30^ Dominican Republic,^31^ Grenada,^32^^,^^33^ and Haiti.^34^

In 2000, an outbreak of NAS occurred in Jamaica that was caused by the consumption of contaminated Caesar salad by 12 visitors.^23^^–^^25^^,^^35^^,^^36^ After these reported human cases, the Jamaica Public Health Department investigated the source of infection. They initiated a study to evaluate the level of *A. cantonensis* infection in natural populations of rats and snails across eight Jamaican sites; they tested 109 rats, of which 24 of 109 (22%) were found to be infected with *A. cantonensis*, and 4 of 48 snails (8%) were found to be infected with L3 larvae.^24^ Around the same time, a lethal NAS case involving a Jamaican infant was caused by *A. cantonensis*, although no initial mechanism of infection could be identified.^25^ An additional study in Jamaica tested 297 *Rattus rattus*, 140 *Rattus norvegicus*, and 777 terrestrial mollusks. This study reported 32% *A. cantonensis* infection in rats and 12.5% *A. cantonensis* infection in mollusks.^35^ In Guadeloupe, an infant was diagnosed with NAS, who likely acquired the larvae from a snail in the home garden; after this, snails tested in the south of the island were found to be infected with *A. cantonensis*.^27^ In the Dominican Republic, a traveler was diagnosed with NAS, likely from eating freshwater shrimp while on holiday.^37^ In Martinique, one of eight NAS cases between 2002 and 2017 resulted in death. In Haiti, concerns over NAS outbreaks on neighboring islands spurred researchers to examine rats for infections with *A. cantonensis*. Of the 44 rats studied, 14 (32%) were infected.^38^ Similarly, in Grenada, of the 192 rats examined for *A. cantonensis* in 2005 and 2006, 45 (23%) were infected.^32^

The ubiquity of NAS in the Caribbean necessitated a study in Puerto Rico. An early survey of rat-hosted helminths in the early 1960s reported the absence of *A. cantonensis* among 108 *R. rattus* and *R. norvegicus* evaluated by necropsy.^39^ However, the parasite was found in several locations by 1984.^40^ To determine infection rates in the definitive hosts, 103 *R. rattus* and* R. norvegicus* were captured between January and April 1984 and evaluated for the presence of *A. cantonensis* in the pulmonary arteries. A total of 10 of 63 *R. rattus* (15.9%) were positive, whereas 19 of 40 *R. norvegicus* (47.5%) were positive. Overall, 29 of 103 rats were infected with *A. cantonensis* (28.2%) from all sites.^40^ These same researchers evaluated the potential risk to human health through contact with snails as intermediate hosts. They collected 611 individual snails from five gastropod species from various sites on the east side of Puerto Rico. Snails were minced and digested in HCl and pepsin, and the filtrate was examined for L3 with light microscopy. Larvae were found in the terrestrial common garden snail *Subulina octona* from five of six sites.^40^ This is the first report of the detection of *A. cantonensis* in Puerto Rico.

Because the global impact of this infectious disease is increasing,^17^^,^^41^ the biogeographic distribution and prevalence of *A. cantonensis* in snails in Puerto Rico need to be reassessed. *Parmarion martensi* was first detected in Puerto Rico in June 2002, and *Parmarion intermedius* was first detected in Puerto Rico in June 2017.^42^ The last published assessment for *A. cantonensis* in Puerto Rico was performed nearly 40 years ago.^40^ Sensitive molecular tools, such as quantitative polymerase chain reaction (qPCR), have since been developed to detect the presence of *A. cantonensis* DNA in gastropods.^43^^–^^46^ The aim of this study was to survey the current prevalence and biogeographic distribution of *A. cantonensis* DNA in multiple gastropod species from eight sites in Puerto Rico by qPCR.

## MATERIALS AND METHODS

### Site selection.

**Figure 1.**
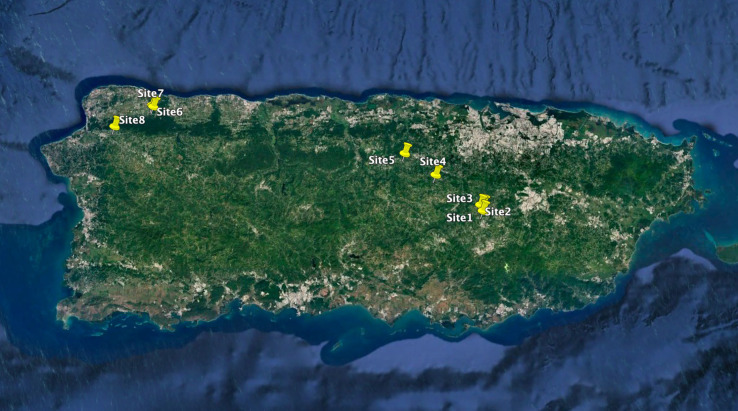
The geographic locations of the eight sample sites across mainland Puerto Rico involved in this study. Longitude, latitude, and site elevation are provided in Table 1. Image courtesy of Google Earth.

Eight locations (Figure 1) on the main island of Puerto Rico were selected by local US Department of Agriculture Animal and Plant Health Inspection Service researchers based on previously observed gastropod populations. Puerto Rico is an 8,870-square-kilometer island with a population of approximately 3.2 million people and a population density of 365 per square kilometer (https://data.census.gov/profile/Puerto_Rico?g=040XX00US72). The proximity of this U.S. territory to Florida and other coastal states in which *A. cantonensis* has already been documented makes Puerto Rico an essential study site.^47^

### Gastropod collection.

**Figure 2.**
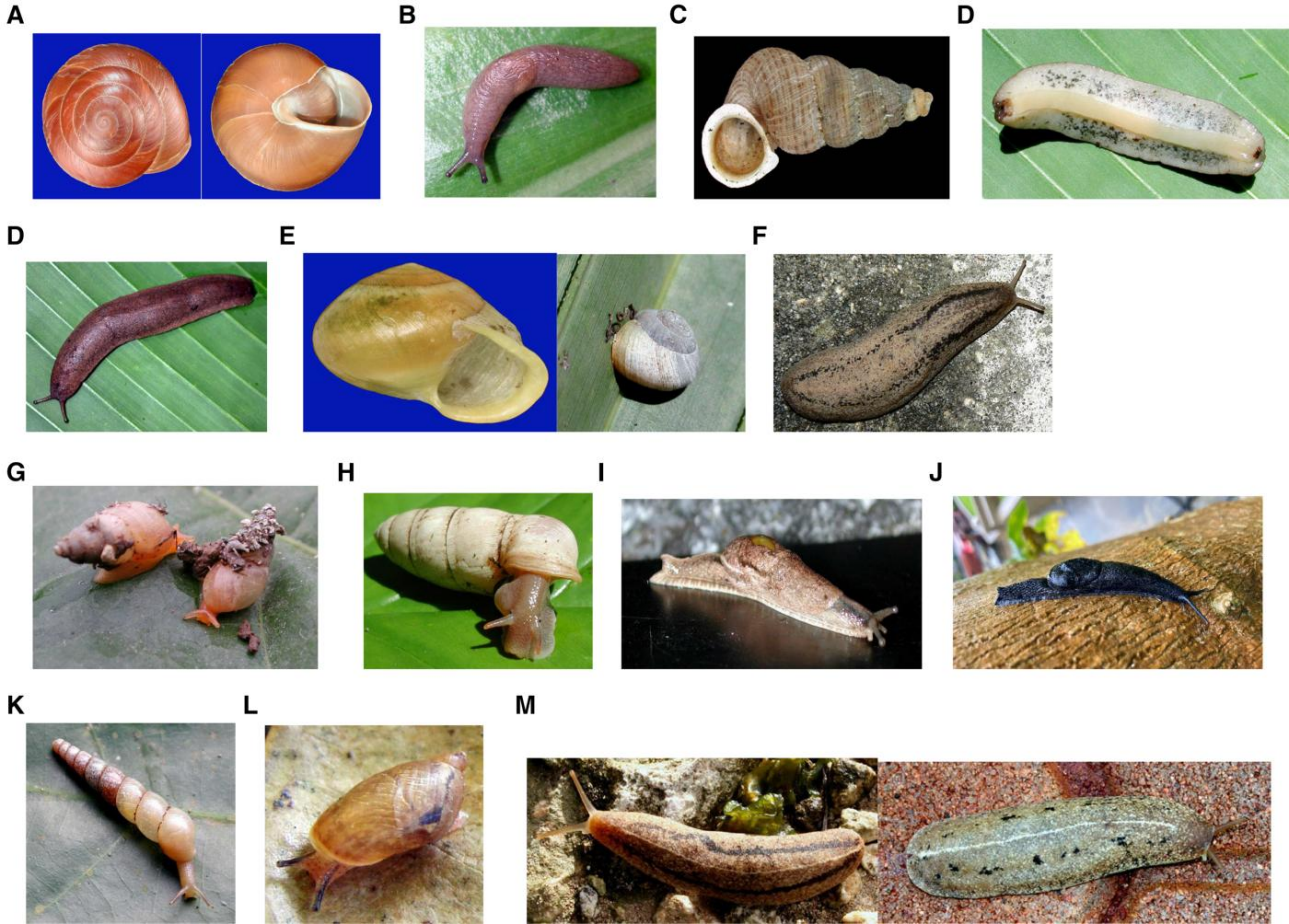
Photographs of the gastropod species collected for this study. (**A**) *Caracolus carocolla* (Linnaeus, 1758); (**B**) *Deroceras laeve* (O. F. Müller, 1774); (**C**) *Diplopoma aguadillensis* (L. Pfeiffer, 1875); (**D**) *Diplosolenodes occidentalis* (Guilding, 1824); (**E**) *Helicina striata* (Lamarck, 1822); (**F**) *Leidyula floridana* (Leidy, 1851); (**G**) *Leptinaria unilamellata* (A. d’Orbigny, 1838); (**H**) *Neopupina croceum* (Gmelin, 1791); (**I**) *Parmarion martensi* (Simroth, 1893); (**J**) *Parmarion intermedius* (Collinge, 1897); (**K**) *Subulina octona* (Bruguière, 1789); (**L**) *Succinea* sp. (Draparnaud, 1801); and (**M**) *Veronicella cubensis* (L. Pfeiffer, 1840). Photos were provided by (**A–H** and **J–M**) David G. Robinson and (**I**) Robert Hollingsworth.

Gastropods (*n* = 205) included in this study were opportunistically collected from eight sites in Puerto Rico between May 1, 2024 and May 7, 2024. Species were identified in the field morphologically and/or by dissection in the laboratory, and many of those are pictured in Figure 2. Gastropods were dissected using instruments sterilized with 50% bleach and thoroughly rinsed in dH_2_O. To reduce contamination, snails were collected with gloved hands at random from various plants and ground locations around each property using inside-out labeled plastic bags (Table 1). Species identification was performed in the field morphologically or in the laboratory through dissection and microscopy. Gastropod tissue samples were transported in 400 *µ*L DNA lysis buffer (0.1 M Tris HCl, 0.1 M ethylenediaminetetraacetic acid, and 2% sodium dodecyl sulfate [SDS]; *n* = 172) or were transported in 180 *µ*L ATL (DNeasy Blood & Tissue Extraction Kit, Qiagen, Valencia, CA; *n* = 33), to which 220 *µ*L of ATL was added to standardize the DNA extraction process. The remainder of the gastropod tissue was stored in the original ziplock bags and frozen. The samples were shipped to Hawai’i for DNA extraction and analysis under Jarvi Laboratory Biological Safety Protocol 2 Permit #205,107,242,024.

**Table 1:** Number of gastropod samples analyzed; site number; latitude, longitude, and date of the sample collection; elevation; and designation of either “high” or “low” elevation

No. of Samples	Site	Latitude	Longitude	Date	Internal Site Name	Elevation (meters)	Elevation Designation
46	1	18.1883	−66.1640	May 1, 2024	Rd #782 Calle Zafiro	532.49	High
20	2	18.2025	−66.1702	May 1, 2024	Rd #782 Banana trees	526.08	High
10	3	18.2025	−66.1640	May 1, 2024	Rd #782 interior	495.3	High
15	4	18.2770	−66.2811	May 2, 2024	Naranjito Farm Cam La Sabana	470.92	High
35	5	18.3272	−66.3590	May 2, 2024	Corozol Ag Station Rd 5568	230.12	High
25	6	18.4457	−66.9954	May 7, 2024	Rd 475 Km Isabela 1	153.62	Low
16	7	18.4423	−66.9927	May 7, 2024	Rd 475 Km Isabela 2	181.36	Low
38	8	18.3943	−67.0901	May 7, 2024	Rd 444 2.0 Km Moca	67.06	Low
205							

The internal site name is the locally common US Department of Agriculture Animal and Plant Health Inspection Service designation for those sites.

### DNA extraction.

All laboratory benchtops, racks, and pipettes were sterilized with DNA Away™ (Thermo Fisher Scientific, Waltham, MA). Reagents (except for proteinase K), racks, and clean tubes were sterilized by ultraviolet light for 30 minutes (UVP CX-2000 UV Crosslinker, UVP Crosslinker Analytik, Jena, Germany) at 100 mJ cm^−2^. DNA extraction was performed using the DNeasy Blood & Tissue Kits (Qiagen) following the manufacturer’s instructions with minor modification. Ten (L3) *A. cantonensis* larvae were used as the positive control samples during the DNA extraction and qPCR process. Because the gastropod tissue mass varied, the DNA extraction reagents were adjusted to suit tissues ranging from 4 mg to 187 mg.^48^ Reagent volumes were determined based on the sample mass, which was calculated based on the difference between the initial sample tube weight and the sample tube weight after the sample was added. All samples were extracted using at least 180 *µ*L ATL (Qiagen) or 400 *µ*L DNA lysis buffer. Per the manufacturer’s protocol, snail tissue weighing 25 mg or less was processed with 20 *µ*L of proteinase K, 200 *µ*L of AL, and 200 *µ*L of ethanol (ETOH). Snail tissue of 26 mg to 56 mg was processed with 44 *µ*L of proteinase K, 400 *µ*L total of ATL or DNA lysis buffer, 444 *µ*L of AL, and 444 *µ*L of ETOH. Snail tissue of 57 mg to 111 mg was processed with an additional 400 *µ*L of ATL, 89 *µ*L of proteinase K, 889 *µ*L AL, and 889 *µ*L ETOH. Snail tissue of 112 mg to 166.7 mg was processed with an additional 800 *µ*L of ATL, 133 *µ*L of proteinase K, 1,333 *µ*L AL, and 1,333 *µ*L ETOH. Lastly, snail tissue of 167 mg to 222 mg was processed with an additional 1,200 *µ*L ATL, 178 *µ*L of proteinase K, 1,778 *µ*L AL, and 1,778 *µ*L ETOH. Two washes of AW1 and AW2 each were performed, and samples were eluted twice; they were incubated with 200 *µ*L of Qiagen Elution Buffer AE at room temperature for 5 minutes and then, centrifuged for 2 minutes at 8,000 rpm (deviating from the manufacturer’s protocol) for each elution. The final DNA elution volume for each sample was 400 *µ*L, and the samples were split into two 0.6-mL tubes (approximately 200 *µ*L per tube).

### Quantitative polymerase chain reaction.

Quantitative polymerase chain reaction was performed using a QuantStudio5™ Real Time Instrument Desktop 144B05 and software v. 1.5.2 (Applied Biosystems, Thermo Fisher Scientific, Singapore). To quantify the *A. cantonensis* larvae DNA per sample, 0.5 *µ*L of primers and probe were added with 0.5 *µ*L deionized water (DNA free water) to 10 *µ*L TaqMan Fast Master Mix (Life Technologies, Vilnius, Lithuania). A total of 9 *µ*L of genomic DNA sample template was used per polymerase chain reaction, resulting in 20-*µ*L reactions. Similarly, 2 *µ*L of genomic DNA (with 7 *µ*L of dH_2_O) was used in generating the standard curve. The AcanR3990 primers (0.5 *µ*M)^6^ used were forward primer: 5′-AAACTGTTGCTTTCGAAGCTATG-3′; reverse primer: 5′-GCGCAAATCTGACGTTCTTG-3′; and probe (0.25 *µ*M): 5′ 6-FAM/ACA TGA AAC/ZEN/ACC TCA AAT GTG CTT CGA/3′ IABkFQ/ labeled with FAM (6-carboxyfluorescein), a reporter dye fluorophore. All reactions were completed in triplicate.

## STATISTICAL ANALYSES

Metadata were organized, and the means, standard deviations (SDs), and standard errors (SEs) were calculated in Microsoft Excel (Microsoft Corp, Redmond, WA) or Google Sheets (Google, Mountain View, CA). Statistical tests and the bubble plot were generated in RStudio v. 4.2.0 using base R statistical functions and ggplot2 (R Foundation, Vienna, Austria).^49^

## RESULTS

Tissue samples from 204 gastropods representing 18 known species and 1 unknown species as well as one planarian (total of 205) were tested by qPCR for the presence of *A. cantonensis* DNA. Of the 205 samples, 73 (35.6%) were positive, 86 (42%) were negative, and 46 (22.4%) were equivocal (undetermined). Samples with parasite DNA detected by qPCR in triplicate are listed as a positive result here with cycle threshold (C_T_) SDs <0.5 and C_T_ values less than 35 (Table 2). Samples considered “equivocal” denote that the C_T_ SD was >0.5, indicating that detected quantities could not be determined with any certainty when assessing duplicates or triplicates. The total lack of *A. cantonensis* DNA detection in all of the triplicates was deemed to be negative.

**Table 2:** Number of samples analyzed per family and per site that yielded positive, negative, or equivocal results

Family	Species	Site 1			Site 2			Site 3			Site 4			Site 5			Site 6			Site 7			Site 8			Total Samples per Species
		+	−	e	+	−	e	+	−	e	+	−	e	+	−	e	+	−	e	+	−	e	+	−	e	
Platyhelminthes	*Bipalium* sp. (Stimpson, 1857)																							1		1
Solaropsidae	*Caracolus carocolla* (Linnaeus, 1758)				3			2																		5
Solaropsidae	*Caracolus marginella* (Gmelin, 1791)	1			2			1			3									1		1				9
Agriolimacidae	*Deroceras laeve* (O. F. Müller, 1774)											1		1	3						1			3		9
Annularidae	*Diplopoma aguadillensis* (L. Pfeiffer, 1875)																		1							1
Veronicellidae	*Diplosolenodes occidentalis* (Guilding, 1824)							1																		1
Sagdidae	*Granodomus lima* (Férussac, 1821)					1	1			2		1	1					4			4	1				15
Helicinidae	*Helicina striata* (Lamarck, 1822)																	1								1
Veronicellidae	*Leidyula floridana* (Leidy, 1851)														2									1		3
Achatinidae	*Leptinaria unilamellata* (A. d’Orbigny, 1838)																							3	2	5
Megalomastomatidae	*Neopupina croceum* (Gmelin, 1791)																0	7	1							8
Philomycidae	*Pallifera* sp. (E. S. Morse, 1864)		1	1	2		3			1	1	1	1		1	1								1		14
Ariophantidae	*Parmarion intermedius* (Collinge, 1897)	26		1				1			3	1		4	5	2								1		44
Ariophantidae	*Parmarion martensi* (Simroth, 1893)	3	2	3	4	1	2	1							1	1							3	2		23
Achatinidae	*Subulina octona* (Bruguière, 1789)																	2			1					3
Succineidae	*Succinea* sp. (Draparnaud, 1801)													1	2	4										7
N/A	Unidentified snail										1								1							2
Veronicellidae	*Veronicella cubensis* (L. Pfeiffer, 1840)									1				3	1			1								6
Veronicellidae	*Veronicella* sp. (Blainville, 1817)												1			2	1	5	1		5		4	9	2	30
Zachrysiidae	*Zachrysia auricoma havanensis* (Pilsbry, 1894)		3	5																	2			5	3	18
	Postive total	30			11			6			8			9			1			1			7			73
	Negative total		6			2			0			4			15			20			13			26		86
	Equivocal/negligible total			10			6			4			3			10			4			2			7	46
	Site and full study totals	46			19			10			15			34			25			16			40			205

e = equivocal; N/A = not applicable. *Angiostrongylus cantonensis* DNA quantification results were by quantitative polymerase chain reaction in triplicate using the AcanR3990 assay.

The species in which *A. cantonensis* DNA was not detected were* Bipalium* sp. (*n* = 1), *Helicina striata* (*n* = 1),* Leidyula floridana* (*n* = 3), and* Subulina octona* (*n* = 3) (Table 2). The only specimen of *Diplopoma aguadillensis*, which was collected from site 6, was equivocal (Table 2).

**Figure 3.**
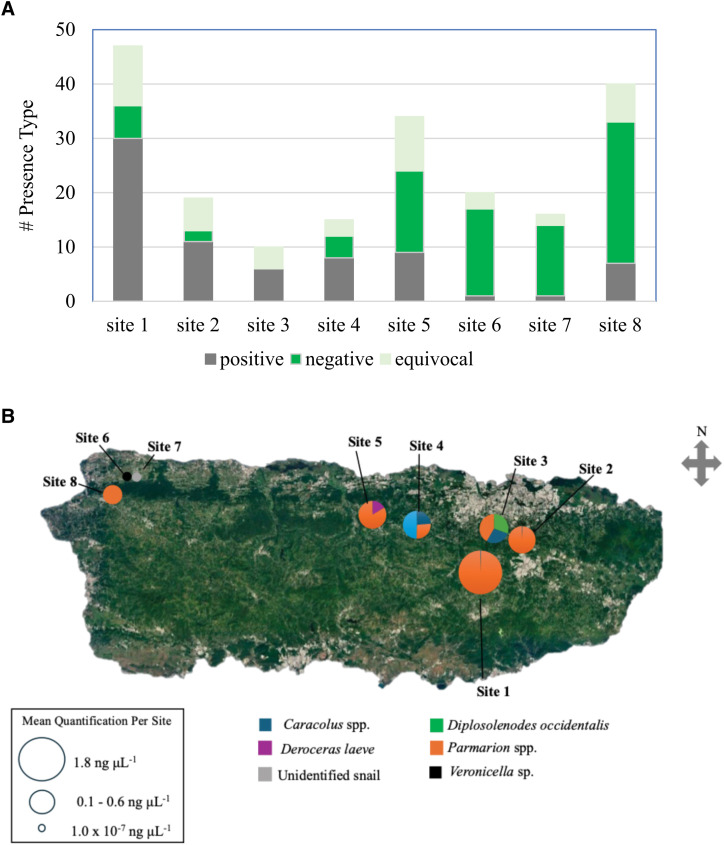
(**A**) Stacked bar plot of the positive (gray), negative (dark green), and equivocal (light green) results based on the AcanR3990 quantitative polymerase chain reaction assay at the eight sites sampled in Puerto Rico in May 2024. (**B**) Map of Puerto Rico with the quantification proportions of *Angiostrongylus cantonensis* DNA detected by qPCR by species and site. The largest circle represents the highest quantification (values of approximately 1.8 ng *µ*L^−1^), the medium-sized bubbles represent the midrange of quantities found in this study (approximately 0.1–0.6 ng *µ*L^−1^), and the lowest concentration (approximately 1.0 × 10^−7^ ng *µ*L^−1^) is represented by the smallest bubbles. For simplification, the proportions of *Caracolus carocolla* and* Caracolus marginella* specimen results per site were combined for this figure as *Caracolus* spp., and* Parmarion intermedius* and *Parmarion martensi* qPCR quantification results were similarly combined and presented under* Parmarion* spp. Species or genera present at each site with the corresponding quantity of *A. cantonensis* DNA are represented by various colors in each pie chart, and they include *Caracolus* spp. (teal),* Deroceras laeve* (purple),* Diplosolenodes occidentalis* (green), *Parmarion* spp. (orange), *Veronicella* sp. (black), and an unidentified snail (gray).

The quantity of* A. cantonensis* DNA found in the gastropods differed by site (Kruskal–Wallis, χ^2^ = 68.404, degrees of freedom [df] = 7, *P* <0.001). *Parmarion* and *Caracolus* gastropods contained a significantly greater concentration of *A. cantonensis* DNA and thus, were suspected to be more highly infected than the other species (Wilcoxon post hoc test) (Figure 3; Table 3). The highest concentrations of *A. cantonensis* DNA were found in the northern middle region of Puerto Rico at site 1 (*n* = 46) (Figure 2). At site 2 (Figure 3; Table 3), *Caracolus marginella* (positive: *n* = 3, negative: *n* = 0) (Table 3) was found to have the highest mean concentration of *A. cantonensis* DNA (5.96 × 10^−3^ ± 2.02 × 10^−3^ ng *µ*L^−1^). The SE is high in several of the averages because some gastropods tested with high concentrations, whereas others of the same species had none or were equivocal (Table 3). *Parmarion intermedius* specimens collected from site 1 (positive: *n* = 26, negative: *n* = 1) yielded an average concentration of 2.320 ± 0.786 ng *µ*L^−1^ of DNA (Table 3). In contrast, site 7 (*n* = 16) and site 8 (*n* = 35) on the northwestern side of Puerto Rico had the lowest concentrations of DNA (Figure 3; Table 3). *Angiostrongylus cantonensis* DNA was detected in one *Veronicella* sp. specimen at site 6 (*n* = 25). The* A. cantonensis* DNA concentrations in the collected gastropod species were significantly different from each other (Kruskal–Wallis, χ^2^ = 101.33, df = 7, *P* <0.01). Additionally, site elevation appeared to play a role, with significant differences detected among all of the sites (Kruskal–Wallis, df = 7, *P* <0.05) and by elevation designation (Table 1) (high elevation being 471–532 meters and low elevation being 0–230 meters, *t*-test *P* <0.0001).

**Table 3:** Mean quantification ± standard error (nanograms per microliter^−1^) of positive *Angiostrongylus cantonensis* DNA concentrations from snail specimens tested in triplicate quantitative polymerase chain reaction testing using the AcanR3990 assay per sample by site

Specimen	Site 1	Site 2	Site 3	Site 4	Site 5	Site 6	Site 7	Site 8	Total No. of Positive Samples per Species
Positive no.		**3**	**2**						**5**
*Caracolus carocolla*		2.23 × 10^−3^ ± 2.23 × −10^−3^	0.255 ± 0.04						
Positive no.	**1**	**2**	**1**	**3**			**1**		**8**
*Caracolus marginella*	0.025	5.96 × 10^−3^ ± 2.02 × 10^−3^	0.53	0.56 ± 0.56			1.00 × 10^−4^		
Positive no.					**1**				**1**
*Deroceras laeve*					0.09 ± 0.05				
Positive no.			**1**						**1**
*Diplosolenodes occidentalis*			0.85						
Positive no.		**2**		**1**					**3**
*Pallifera* sp.		3.12 × 10^−6^ ± 2.92 × 10^−6^		4.81 × 10^−7^± 4.81 × 10^−7^					
Positive no.	**26**		**1**	**3**	**4**				**34**
*Parmarion intermedius*	2.32 ± 0.79		1.13	0.62 ± 0.44	0.49 ± 0.15				
Positive no.	**3**	**4**	**1**					**3**	**11**
*Parmarion martensi*	3.00 ± 1.99	1.00 ± 1.00	0.036					6.26 × 10^−2^ ± 3.76 × 10^−2^	
Positive no.					**1**				**1**
*Succinea* sp.					3.22 × 10^−7^				
Positive no.				**1**					**1**
Unidentified snail				1.16					
Positive no.					**3**				**3**
*Veronicella cubensis*					9.15 × 10^−8^ ± 5.01 × 10^−8^				
Positive no.						**1**		**4**	**5**
*Veronicella* sp.						2.59 × 10^−7^ ± 2.57 × 10^−7^		1.93 × 10^−7^ ± 1.14 × 10^−7^	
Total no. of positive samples per site	30	11	6	8	9	1	1	7	73

The bold numbers are the total number of samples testing positive (bold), with the mean DNA concentrations of samples reported after PCR.

## DISCUSSION

The highest prevalence of *A. cantonensis* DNA was found in the *Parmarion* and* Caracolus* genera at multiple sites (Figure 3; Table 3), with site 4 and site 5 exhibiting the highest concentrations. The extremely low concentrations of DNA found in the samples from some individuals may be because of crosscontamination by slime trail or interactions with other gastropods on site, whereby individuals may pick up larval DNA fragments from other gastropods carrying larger quantities of *A. cantonensis*.^50^^,^^51^ This may explain why some individuals yielded very little DNA or were equivocal.

When compared with other studies of *A. cantonensis* in the Caribbean, our results show differing infection levels among certain species. Research elsewhere in Latin America shows that the Veronicellid slugs (e.g., *Veronicella*, *Leidyula*, etc.) are major carriers of *A. cantonensis*,^52^ which is not reflected in this study, likely because of the small sampling sizes. Of the *Veronicella* sp. individuals testing positive, 17% (*n* = 5/30) were collected from the lower-elevation northwestern sites (site 6 and site 8) (Figure 3; Tables 2 and 3). At site 5, half (*n* = 3/6 or 50%) of the study total *Veronicella cubensis* individuals were positive (Table 2). All *Leidyula floridana* (*n* = 3) specimens yielded equivocal results, which warrants more testing across various sites (Table 2).

Both *V*. *cubensis* and an unidentified *Veronicella* species were collected at site 6 (*n* = 8) in the northwestern part of Puerto Rico, where *A. cantonensis* DNA was detected in one specimen of *Veronicella* (Figure 3; Table 2). Most of the Veronicellidae collected in this study were negative for *A. cantonensis* or equivocal, including *Veronicella* sp. (*n* = 25/30 or 83.3%), *V. cubensis* (*n* = 3/6 or 50%), and *L. floridana* (*n* = 3/3 or 100%).

*Parmarion martensi* was introduced to Puerto Rico in 2002,^42^ and *Parmarion* spp. were found at all sites except site 6 and site 7; 45 of 67 (67.2%) were positive. The interactions between snails and humans may be facilitated by the recognized climbing abilities of *P. martensi* as is observed in Hawai’i, and *P. martensi* is documented as having one of the highest infection rates in Hawai’i.^19^ At night, they can climb up home walls, plastic drainpipes, water tanks, decks, stairs (wood), sinks, tarps, black plastic sheeting, and plant pots, and they have even been reported crawling on a toothbrush left outside.^19^ During the day, they generally prefer to remain in dark, moist places, like under rocks, and in covered areas with plant material, such as mulch, compost, and fallen leaves or fronds. Not only do they feed on plant material, but also, they have been recorded to consume pet food, hibiscus flowers, and ripe papayas still on the tree.^19^ Snails on vegetables or in water may transmit larvae unknowingly to people.^11^^,^^53^ Therefore, a behavioral study on the movements of *Parmarion* in Puerto Rico would elucidate the potential interactions between humans and *P. martensi* as a vector for rat lungworm disease.

*Caracolus carocolla* are frequently found 0 to 3 meters from the ground in trees or on the ground, and they depend on a diet of diatoms, wood cells, plant hairs, leaf cells, and mycelia. They can live for 15 years and hermaphroditically mate throughout the year.^54^^–^^56^ Alvarez and Willig^55^ found that *C. carocolla* was more abundant in moist areas, such as leaf litter, which was also reported by Heatwole and Heatwole.^54^ Previous studies have shown that *C. carocolla* was the most abundant snail species found in the Luquillo Experimental Forest area located in Puerto Rico.^57^ Here, the snails may have been out in the open (on trees and in leaf litter) because it had been raining more than usual during the weeks before collection (Climate and Hydrology Monthly Report for Puerto Rico and the US Virgin Islands; https://www.weather.gov/media/sju/climo/monthly_reports/2024/Apr2024.pdf). Both* C. carocolla* and *R. rattus* are arboreal, and they may overlap in forest ranges during wet seasons; all five samples tested were positive. Interestingly, more *Carocolus* spp. (Solaropsidae) and *Parmarion* spp. (Ariopanthidae) in number and in *A. cantonensis* DNA quantities were found at the higher-elevation and more-central sites (sites 1–4), whereas more Veronicellidae snails were found at the lower-elevation sites (sites 6–8) on the northwestern side of Puerto Rico.

The common garden snail *Subulina octona* had been known as a carrier of *A. cantonensis* since the early 1960s.^58^ de León^39^ examined rats and *S. octona* for the presence of *A. cantonensis*, yet none were found in 1964 over a 6-month period in Puerto Rico. However, Andersen et al.^40^ showed *S. octona* to have been infected with *A. cantonensis* in five of six sites on the east side of Puerto Rico. The reported infection rate was 42%, which differs from our findings as none of the *S. octona* (*n* = 3) (Table 2) collected and tested in this study were infected. Andersen et al.^40^ also tested *R. rattus* and* R. norvegicus* for the presence of *A. cantonensis* in the pulmonary arteries. From all testing sites, 29 of 103 rats were infected with *A. cantonensis* (28.2%), which included 16% of* R. rattus* and 48% of *R. norvegicus.*^40^

Gastropod community structure in Puerto Rico is determined by environmental conditions, including more specifically, plant assemblages.^59^ Changes in elevation, soil, floral communities, and predators impact the land snail *Helicina striata*, *C. caracolla*, and *C. marginella* populations.^59^ During rainy seasons, there is an increased chance of snail–human interactions as evidenced by six of eight cases of human infection between 2002 and 2015 in Martinique occurring during rainy seasons.^26^ Rainfall and elevation may play a role in the abundance of *Parmarion* and *Caracolus*, the two genera with the highest concentration of *A. cantonensis* DNA, given that rainfall in the month prior (April 2024) was higher than normal. Additionally, pest treatment methods may differ among sites, impacting the abundance of rats and slugs carrying *A. cantonensis*.

*Parmarion* and* Caracolus* spp. are the main gastropod carriers of *A. cantonensis* among the species tested in Puerto Rico. These species may be of special concern to farmers, community members, and resource managers, particularly during seasons of high rainfall. Puerto Rico is only ∼1,022 miles (∼1,645 kilometers) from Florida, and there is concern that the two *Parmarion* species now known to exist in Puerto Rico could potentially be introduced to Florida, where semislugs have not yet been detected. This is the scenario that previously occurred in Hawai’i, resulting in increased spread of the parasite and increases in cases of human infection. The *A. cantonensis* parasite itself is documented all throughout the southeastern United States, and* R. rattus* and *A. cantonensis* have been documented in the Miami area.^46^ Surveys in Florida^47^ evaluated two species and one other genus that were also evaluated in this study. These researchers found *Helicina striata* and *Zachrysia* spp. samples to carry *A. cantonensis* DNA using polymerase chain reaction, whereas *Subulina octona* yielded negative results; however, our results showed negative results for *H. striata* (*n* = 1), *S. octona* (*n* = 3), and *Zachrysia* spp. (*n* = 18). Based on these findings, DNA testing for the presence of *A. cantonensis* throughout Puerto Rico at varying elevations during different seasons would be prudent to further evaluate the biogeography of the parasite.

## CONCLUSION

This is the largest study to assess levels of gastropods with *A. cantonensis* DNA that has been done in Puerto Rico since Andersen et al.^40^ in 1986. Our preliminary work sheds light on the importance of testing for rat lungworm disease, the need for clinical guidelines and awareness among hospital staff, and the need for the inclusion of recently published NAS guidelines in cases of suspected exposure or ingestion. To help prevent human cases, public warnings against consuming raw or undercooked snails may be helpful. Although some snails in this study hosted large parasite loads, this work represents an exploratory overview of eight sites in Puerto Rico. Because *A. cantonensis* can cause serious neurological damage to humans and some domestic animals,^4^^,^^60^ monitoring for changes in snail populations over time is prudent. Future studies might consider including examining the history of human NAS cases in Puerto Rico as no literature was found on this subject at the time of this publication.

## References

[1] Chen HT, 1935. Un nouveau nématode pulmonaire, *Pulmonema cantonensis*, n g., n. sp. *Annales de Parasitologie Humaine et Comparée* 13(4): 312–317.

[2] Rosen L Chappell R Laqueur GL Wallace GD Weinstein PP, 1962. Eosinophilic meningoencephalitis caused by a metastrongylid lung-worm of rats. JAMA 179(8): 620–624. 14493905 10.1001/jama.1962.03050080032007

[3] Mackerras M Sandars G, 1955. The life history of the rat lungworm, *Angiostrongylus cantonensis* (Chen) (Nematoda: Metastrongylidae). Aust J Zool 3(1): 1–21.

[4] Jarvi S Jacob J Mina A Lyons M, 2024. Detection of rat lungworm (*Angiostrongylus Cantonensis*) infection by real-time PCR from the peripheral blood of animals: A preliminary study. Parasitol Res 123(6): 240. 38862687 10.1007/s00436-024-08251-9 PMC11166865

[5] Turck HC Fox MT Cowie RH, 2022. Paratenic hosts of *Angiostrongylus cantonensis* and their relation to human neuroangiostrongyliasis globally. One Health 15: 100426. 36277113 10.1016/j.onehlt.2022.100426 PMC9582568

[6] Sears WJ, , 2021. AcanR3990 qPCR: A novel, highly sensitive, bioinformatically-informed assay to detect *Angiostrongylus cantonensis* infections. Clin Infect Dis 73(7): e1594–e1600. 33252651 10.1093/cid/ciaa1791 PMC8492198

[7] Costa LRR McClure JJ Snider TG III Stewart TB, 2000. Verminous meningo-encephalomyelitis by *Angiostrongylus (=Parastrongylus) cantonensis* in an American Miniature Horse. Equine Vet Educ 12(1): 2–6.

[8] Gardiner CH Wells S Gutter AE Fitzgerald L Anderson DC Harris RK Nichols DK, 1990. Eosinophilic meningoencephalitis due to *Angiostrongylus cantonensis* as the cause of death in captive non-human primates. Am J Trop Med Hyg 42(1): 70–74. 2301708 10.4269/ajtmh.1990.42.70

[9] Niebuhr CN Jarvi SI Kaluna L Fischer BLT Deane AR Leinbach IL Siers SR, 2020. Occurrence of rat lungworm (*Angiostrongylus cantonensis*) in invasive coqui frogs (*Eleutherodactylus Coqui*) and other hosts in Hawaii. J Wildl Dis 56(1): 203–207. 31295084

[10] Cross J, 2004. *Angiostrongylus (Parastrongylus) cantonensis* in the western hemisphere. Southeast Asian J Trop Med Public Health 35(1): 107–111.

[11] Howe K Kaluna L Lozano A Torres Fischer B Tagami Y McHugh R Jarvi S, 2019. Water transmission potential of *Angiostrongylus cantonensis*: Larval viability and effectiveness of rainwater catchment sediment filters. PLoS One 14(4): e0209813. 31022202 10.1371/journal.pone.0209813 PMC6483183

[12] Alicata JE, 1965. Biology and distribution of the rat lungworm, *Angiostrongylus cantonensis,* and its relationship to eosinophilic meningoencephalitis and other neurological disorders of man and animals. Adv Parasitol 3: 223–248. 5334821 10.1016/s0065-308x(08)60366-8

[13] Kliks MM Palumbo NE, 1992. Eosinophilic meningitis beyond the Pacific Basin: The global dispersal of a peridomestic zoonosis caused by *Angiostrongylus cantonensis*, the nematode lungworm of rats. Soc Sci Med 34(2): 199–212. 1738873 10.1016/0277-9536(92)90097-a

[14] Prociv P Turner M, 2018. Neuroangiostrongyliasis: The “subarachnoid phase” and its implications for anthelminthic therapy. Am J Trop Med Hyg 98(2): 353–359. 29210355 10.4269/ajtmh.17-0206 PMC5929180

[15] Jarvi S Prociv P, 2021. *Angiostrongylus cantonensis* and neuroangiostrongyliasis (rat lungworm disease) 2020. Parasitology 148(2): 129–132. 33315004 10.1017/S003118202000236X PMC11010204

[16] Ansdell V, , 2021. Guidelines for the diagnosis and treatment of neuroangiostrongyliasis: Updated recommendations. Parasitology 148(2): 227–233. 32729438 10.1017/S0031182020001262 PMC7887556

[17] Cowie RH Ansdell V Panosian Dunavan C Rollins RL, 2022. Neuroangiostrongyliasis: Global spread of an emerging tropical disease. Am J Trop Med Hyg 107(6): 1166–1172. 36343594 10.4269/ajtmh.22-0360 PMC9768254

[18] Gottdenker NL, , 2023. *Angiostrongylus cantonensis* infection in brown rats (*Rattus norvegicus*), Atlanta, Georgia, USA, 2019–2022. Emerg Infect Dis 29(10): 2167–2170. 37735783 10.3201/eid2910.230706 PMC10521602

[19] Hollingsworth RG Kaneta R Sullivan JJ Bishop HS Qvarnstrom Y Da Silva AJ Robinson DG, 2007. Distribution of *Parmarion* cf. *martensi* (Pulmonata: Helicarionidae), a new semi-slug pest on Hawai ‘i Island, and its potential as a vector for human angiostrongyliasis. Pac Sci 61(4): 457–467.

[20] Cowie RH Hayes KA Kim JR Bustamente KM Yeung NW, 2018. Parmarion martensi Simroth, 1893 (Gastropoda: Ariophantidae), an intermediate host of Angiostrongylus cantonensis (rat lungworm), on Maui. Bishop Museum Occas Pap 123: 7–10.

[21] Howe K Jarvi SI, 2017. Angiostrongyliasis (rat lungworm disease): Viewpoints from Hawaii Island. ACS Chem Neurosci 8(9): 1820–1822. 28820576 10.1021/acschemneuro.7b00299

[22] Chance MD, , 2024. *Angiostrongylus cantonensis* meningoencephalitis in three pediatric patients in Florida, USA. J Pediatric Infect Dis Soc 13(12): 639–642. 39529387 10.1093/jpids/piae113 PMC11717585

[23] Slom TJ, , 2002. An outbreak of eosinophilic meningitis caused by angiostrongylus cantonensis in travelers returning from the Caribbean. N Engl J Med 346(9): 668–675. 11870244 10.1056/NEJMoa012462

[24] Lindo JF, , 2002. Enzootic *Angiostrongylus cantonensis* in rats and snails after an outbreak of human eosinophilic meningitis, Jamaica. Emerg Infect Dis 8(3): 324–326. 11927033 10.3201/eid0803.010316 PMC2732477

[25] Lindo JF Escoffery CT Reid B Codrington G Cunningham-Myrie C Eberhard ML, 2004. Fatal autochthonous eosinophilic meningitis in a Jamaican child caused by *Angiostrongylus cantonensis*. Am J Trop Med Hyg 70(4): 425–428. 15100458

[26] Dard C Tessier E Nguyen D Epelboin L Harrois D Swale C Cabié A de Meuron K Miossec C Desbois-Nogard N, 2020. First cases of *Angiostrongylus cantonensis* infection reported in Martinique, 2002–2017. Parasite 27(May): 31. 32394891 10.1051/parasite/2020032 PMC7216674

[27] Dard C Piloquet JE Qvarnstrom Y Fox LM M’kada H Hebert JC Mattera D Harrois D, 2017. First evidence of angiostrongyliasis caused by in Guadeloupe, Lesser Antilles. Am J Trop Med Hyg 96(3): 692–697. 28070007 10.4269/ajtmh.16-0792 PMC5361547

[28] Martínez-Delgado JF González-Cortiñas M Tápanes-Cruz TR Ruiz-Méndez A, 2000. Eosinophilic meningoencephalitis in Villa Clara (Cuba). A study of 17 patients. Rev Neurol 31(5): 417–421. 11027091

[29] Aguiar PH Morera P Pascual J, 1981. First record of *Angiostrongylus cantonensis* in Cuba. Am J Trop Med Hyg 30(5): 963–965. 7283015 10.4269/ajtmh.1981.30.963

[30] Jaume ML Perera de Puga G Aguiar Prieto PH, 1981. Bradybaena similaris (Ferussac): Intermediate host of *Angiostrongylus cantonensis* in Cuba. Rev Cubana Med Trop 33(3): 207–209. 7038796

[31] Vargas MJ Gomez Perez D Malek EA, 1992. First record of *Angiostrongylus cantonensis* (Chen, 1935) (Nematoda: Metastrongylidae) in the Dominican Republic. Trop Med Parasitol 43(4): 253–255. 1293731

[32] Chikweto A Bhaiyat MI Macpherson CNL Deallie C Pinckney RD Richards C Sharma RN, 2009. Existence of *Angiostrongylus cantonensis* in rats (*Rattus Norvegicus*) in Grenada, West Indies. Vet Parasitol 162(1–2): 160–162. 19304395 10.1016/j.vetpar.2009.02.020

[33] Coomansingh-Springer C Vishakha V Montanez Acuna A Armstrong E Sharma RN, 2019. Internal parasitic burdens in brown rats (*Rattus norvegicus*) from Grenada, West Indies. Heliyon 5(8): e02382. 31517102 10.1016/j.heliyon.2019.e02382 PMC6728764

[34] Fedna J Borne R Rieffel D Bornette G Henrys JH Grenouillet F Raoul F, 2024. Molecular study of the status of *Angiostrongylus cantonensis* in rats in Haiti. Parasite 31: 64. 39853112 10.1051/parasite/2024063 PMC11465708

[35] Waugh CA Lindo JF Lorenzo-Morales J Robinson RD, 2016. An epidemiological study of *A. cantonensis* in Jamaica subsequent to an outbreak of human cases of eosinophilic meningitis in 2000. Parasitology 143(9): 1211–1217. 27350332 10.1017/S0031182016000640

[36] Waugh CA Shafir S Wise M Robinson RD Eberhard ML Lindo JF, 2005. Human *Angiostrongylus cantonensis*, Jamaica. Emerg Infect Dis 11(12): 1977–1978. 16485498 10.3201/eid1112.050217 PMC3367624

[37] Leone S De Marco M Ghirga P Nicastri E Esposito M Narciso P, 2007. Eosinophilic meningitis in a returned traveler from Santo Domingo: Case report and review. J Travel Med 14(6): 407–410. 17995537 10.1111/j.1708-8305.2007.00152.x

[38] Raccurt CP Blaise J Durette‐Desset MC, 2003. Présence d’ *Angiostrongylus cantonensis* en Haïti. Tropical Med Int Health 8(5): 423–426. 10.1046/j.1365-3156.2003.01035.x 12753637

[39] de León DD, 1964. No *Angiostrongylus cantonensis* (Nematoda: Metastrongylidae) observed in 108 rats in San Juan P.R. J Agric Univ Puerto Rico 48(4): 353–354.

[40] Andersen E Gubler DJ Sorensen K Beddard J Ash LR, 1986. First report of *Angiostrongylus cantonensis* in Puerto Rico. Am J Trop Med Hyg 35(2): 319–322. 3953946 10.4269/ajtmh.1986.35.319

[41] Barratt J Chan D Sandaradura I Malik R Spielman D Lee R Marriott D Harkness J Ellis J Stark D, 2016. *Angiostrongylus cantonensis*: A review of its distribution, molecular biology and clinical significance as a human pathogen. Parasitology 143(9): 1087–1118. 27225800 10.1017/S0031182016000652

[42] Robinson DG, , 2025. On the occurrence of two Southeast Asian semi-slugs in Puerto Rico. (In prep).

[43] Qvarnstrom Y Sullivan JJ Bishop HS Hollingsworth R da Silva AJ, 2007. PCR-based detection of *Angiostrongylus cantonensis* in tissue and mucus secretions from molluscan hosts. Appl Environ Microbiol 73(5): 1415–1419. 17194836 10.1128/AEM.01968-06 PMC1828786

[44] Qvarnstrom Y, , 2016. Real-time polymerase chain reaction detection of *Angiostrongylus cantonensis* DNA in cerebrospinal fluid from patients with eosinophilic meningitis. Am J Trop Med Hyg 94(1): 176–181. 26526920 10.4269/ajtmh.15-0146 PMC4710426

[45] Jarvi SI Farias MEM Howe K Jacquier S Hollingsworth R Pitt W, 2012. Quantitative PCR estimates *Angiostrongylus cantonensis* (rat lungworm) infection levels in semi-slugs (*Parmarion Martensi*). Mol Biochem Parasitol 185(2): 174–176. 22902292 10.1016/j.molbiopara.2012.08.002 PMC3753181

[46] Stockdale Walden HD Slapcinsky JD Roff S Calle JM Goodwin ZD Stern J Corlett R Conway J McIntosh A, 2017. Geographic distribution of *Angiostrongylus Cantonensis* in wild rats (*Rattus rattus*) and terrestrial snails in Florida, USA. PLoS One 12(5): e0177910. 28542310 10.1371/journal.pone.0177910 PMC5436845

[47] Stockdale-Walden HD Slapcinsky J Qvarnstrom Y McIntosh A Bishop HS Rosseland B, 2015. *Angiostrongylus cantonensis* in introduced gastropods in southern Florida source. J Parasitol 101(2): 156–159. 25564891 10.1645/14-553.1

[48] Niebuhr CN Siers SR Leinbach IL Kaluna LM Jarvi SI, 2021. Variation in *Angiostrongylus cantonensis* infection in definitive and intermediate hosts in Hawaii, a global hotspot of rat lungworm disease. Parasitology 148(2): 133–142. 32907654 10.1017/S003118202000164X PMC11010199

[49] Wickham H, 2009. ggplot2: Elegant Graphics for Data Analysis (Springer Science & Business Media, New York).

[50] Modrý D Fecková B Putnová B Manalo SM Otranto D, 2021. Alternative pathways in (Metastrongyloidea: Angiostrongylidae) transmission. Parasitology 148(2): 167–173. 32981541 10.1017/S0031182020001857 PMC11010052

[51] Rollins RL Medeiros MC Cowie RH, 2023. Stressed snails release *Angiostrongylus cantonensis* (rat lungworm) larvae in their slime. One Health 17: 100658. 38116454 10.1016/j.onehlt.2023.100658 PMC10728333

[52] Robinson DG Fields A, 2010. The leatherleaf slugs (family Veronicellidae). *Regional Workshop Mollusk Pests of Economic Importance*: Centro Kellog, Escuela Agricola Panamericana, Zamorano, Honduras.

[53] Steel A Jacob J Klasner I Howe K Jacquier SH Pitt WC Hollingsworth R Jarvi SI, 2021. In vitro comparison of treatments and commercially available solutions on mortality of *Angiostrongylus cantonensis* third-stage larvae. Parasitology 148(2): 212–220. 32951629 10.1017/S0031182020001730 PMC11010055

[54] Heatwole H Heatwole A, 1978. Ecology of the Puerto Rican camaenid tree-snails. Malacologia 17(2): 241–315.

[55] Alvarez J Willig MR, 1993. Effects of treefall gaps on the density of land snails in the Luquillo Experimental Forest of Puerto Rico. Biotropica 25(1): 100–110.

[56] Cary JF, 1992. *Habitat Selection, Home Range, and Population Dynamics of* Caracolus *c*aracolla *in the Luquillo Experimental Forest of Puerto Rico*. Available at: https://ttu-ir.tdl.org/server/api/core/bitstreams/ed978bb3-9d9d-4979-88aa-fb9195e36e6d/content. Accessed May 6, 2026.

[57] Willig MR Sandlin EA Gannon MR, 1998. Structural and taxonomic correlates of habitat selection by a Puerto Rican land snail. Southwest Nat 43(1): 70–79.

[58] Ash LR, 1962. The helminth parasites of rats in Hawaii and the description of *Capillaria traverae* sp. n. J Parasitol 48(1): 66–68. 13862800

[59] Presley SJ Willig MR Bloch CP Castro-Arellano I Higgins CL Klingbeil BT, 2011. A complex metacommunity structure for gastropods along an elevational gradient. Biotropica 43(4): 480–488.

[60] Jarvi SI, , 2020. Estimating human exposure to rat lungworm (*Angiostrongylus cantonensis)* on Hawai’i Island: A pilot study. Am J Trop Med Hyg 102(1): 69–77. 31769399 10.4269/ajtmh.18-0242 PMC6947786

